# Machine learning modeling for solubility prediction of recombinant antibody fragment in four different *E. coli* strains

**DOI:** 10.1038/s41598-022-09500-6

**Published:** 2022-03-31

**Authors:** Atieh Hashemi, Majid Basafa, Aidin Behravan

**Affiliations:** Department of Pharmaceutical Biotechnology, School of Pharmacy, Shahid Beheshti University of Medical Sciences, ValiAsr Avenue, Niayesh Junction, PO Box 14155-6153, Tehran, Iran

**Keywords:** Applied microbiology, Industrial microbiology

## Abstract

The solubility of proteins is usually a necessity for their functioning. Recently an emergence of machine learning approaches as trained alternatives to statistical models has been evidenced for empirical modeling and optimization. Here, soluble production of anti-EpCAM extracellular domain (EpEx) single chain variable fragment (scFv) antibody was modeled and optimized as a function of four literature based numerical factors (post-induction temperature, post-induction time, cell density of induction time, and inducer concentration) and one categorical variable using artificial neural network (ANN) and response surface methodology (RSM). Models were established by the CCD experimental data derived from 232 separate experiments. The concentration of soluble scFv reached 112.4 mg/L at the optimum condition and strain (induction at cell density 0.6 with 0.4 mM IPTG for 24 h at 23 °C in Origami). The predicted value obtained by ANN for the response (106.1 mg/L) was closer to the experimental result than that obtained by RSM (97.9 mg/L), which again confirmed a higher accuracy of ANN model. To the author’s knowledge this is the first report on comparison of ANN and RSM in statistical optimization of fermentation conditions of *E.coli* for the soluble production of recombinant scFv.

## Introduction

Due to its numerous advantages such as the availability of different genome engineering tools and strategies, established high cell density culture techniques, high growth rate and low protease, *E. coli* has been widely utilized as one of the most favoured microbial hosts for the production of recombinant proteins. Process development and cell engineering are two strategies widely employed to enhance heterologous protein production in this host^1^. The development of production conditions is one of the most influential steps in process development. To obtain best possible production conditions, optimizing variables based on “one-factor-at-a-time” approach in addition to being a labour-intensive process, it is not able to identify interactions between the various parameters involved. Statistical-based and artificial intelligence-based approaches can overcome limitations of the conventional single parametric optimization methods^2^. Response Surface Methodology (RSM) is an efficient optimization method extensively utilized to establish the quantitative relationship between the independent process parameters and responses. Moreover, in RSM, the effects of the variables alone or in combination can be analyzed via regression analysis. Optimum levels of process parameters for preferable responses are also robustly predicted in this method^3^. RSM combined to central composite design has been widely employed in optimization of culture conditions^4^. However, RSM is unable to accurately model a highly non-linear complex system. So, a limited range of input process parameters can be exactly modeled by RSM. Machine learning techniques such as artificial neural network (ANN), which is popular for non-linear multivariate modeling can successfully overcome this limitation of RSM and can be a promising tool for modeling of the biological systems^5^. However, according to their structure, ANN requires processors with parallel processing power. Moreover, there are no specific rules for determining the structure of artificial neural networks. Proper network structure is achieved through trial and error^6^. The most popular ANN network is organized in three layers comprised of input layer, output layer and hidden layer. Different number of hidden layers can be found within a feedforward network^7^. The particular weights of the produced output data by the model are utilized to predict the new set of input data. By presenting sets of input/output data pairs to the neural network, ANN models can be trained. After being trained on the model, the network can correctly predict the outputs corresponding to responses it never has seen before^8^. This approach was successfully utilized as a data analysis tool in fermentation optimization like production of L-asparaginase from *Aspergillus niger*^9^. Several reports have shown that ANN models can work better than RSM when the same DOE has been used. For example Bas and Boyaci results showed the superiority of ANN over RSM in enzyme kinetics^10^.

The optimal conditions for fermentative production of soluble anti- EpCAM extracellular domain (EpEx) single chain variable fragments (scFv) were evaluated in the current study. The scFv represents a class of antibody fragments which is comprised of a heavy chain variable domain (VH) and a light chain variable domain (VL) of an antibody joined by a flexible peptide linker. Its molecular weight is considerably smaller than the full-length antibodies. Owing to small size and low immunogenicity, scFv has brought much attention in biomedicine for theranostic purposes^11^. 4D5MOC-B scFv is a stable anti EpCAM extracellular domain-scFv (anti EpEX-scFv) with a very high affinity to its target. It was generated from the binding residues of parental hybridoma MOC31 which was grafted onto the scFv 4D5 framework. EpCAM was one of the first target antigens considered for tumor immunotherapy because of its overexpression in epithelial-derived neoplasms^12^.

For the first time, this study adopted ANN and RSM to model the effects of post-induction temperature, post-induction time, cell density of induction time, and inducer concentration as numerical factors along with different strains as a categorical factor on soluble production of scFv. Here, the ANN was developed with a large number of experimental data points (232), which reduces problems with overfitting and allows more complex models to be used. Moreover, the optimum culture condition and strain recommended by model were experimentally verified.

## Materials and methods

### Bacterial strains and plasmid

Four *E. coli* strains including SHuffle T7 (gifted by Dr. Nematollahi, Pasteur institute of IRAN, Tehran, Iran), BW25113 (rrnB3 ΔlacZ4787 hsdR514 Δ(araBAD)567 Δ(rhaBAD)568 rph-1 γ (DE3), gifted from Prof. Dr. Silke Leimkühler, University of Potsdam, Potsdam, Germany), Origami (DE3) (Pasteur institute of IRAN, Tehran, Iran), and BL21 (DE3) (gifted by Dr. Keramati, Pasteur institute of IRAN, Tehran, Iran) were used here as the host for antiEpEX-scFv expression. Heat shock method was used to transform the pETDuet-1 plasmid (gifted from Dr. Bandehpour, Shahid Beheshti University of Medical Sciences, Tehran, Iran) containing the antiEpEX-scFv gene into the chemically competent cells of each strain^12^.

### Analytical methods

#### Protein expression

For initial determination of the anti EpEX-scFv expression. Several transformed clones were checked from each strain for their ability to protein expression in similar condition (37 °C, OD 0.8, IPTG 0.8 mM, and 24 h) in 50 mL TY2x medium and results were confirmed by western blotting. In order to perform the optimization experiments, *E. coli* cells were firstly pre-cultured in liquid TY2x medium supplemented with 100 µg/mL ampicillin overnight at 37 °C. Then, 50 mL of medium was inoculated with 10% (v⁄v) of the pre-culture. This culture was used for all experiments designed by RSM-CCD methodology.

#### Sample preparation

After centrifugation of culture medium (10,000 g for 10 min at 4 °C), the cell pellets were resuspended in 20 mL of lysis buffer containing 1 mg/mL lysozyme, 20 mM Tris pH 7.5, 50 mM NaCl and 50% glycerol followed by incubation on ice for 40 min. the cells were then sonicated for 20 min (20 s on/3 s off) at 400 W and centrifuged at 4 °C (15,000 × g for 30 min). The obtained supernatants and pellets were collected as soluble and insoluble fractions respectively.

#### SDS-PAGE and expression analysis

The expression level of the recombinant protein was analyzed utilizing sodium dodecyl sulfate polyacrylamide gel electrophoresis (SDS- PAGE). The samples were resuspended in 4 × SDS sample buffer. After heating at 100 °C for 5 min, 10 μL of each sample was loaded onto 15% SDS-PAGE gel and electrophoresis was carried out. The protein bands were detected via staining the gel with coomassie brilliant blue G-250 staining solution. The signal intensities of the protein bands in all 120 PAGEs were densitometrically determined utilizing ImageJ software (NIH, MD).

#### Western blotting

After separation, protein bands were transferred from a SDS- PAGE gel onto the polyvinylidene difluoride (PVDF) membrane using electroblotting (wet Transblot, Bio-Rad, USA). After blocking in 5% non-fat milk in tris-buffered saline-tween (TBST) for 1 h, the transferred membrane was washed with TBST for three times and incubated overnight with anti-6 × His tag antibody (Sigma, UK). Then, the membrane was washed by TBST three times and incubated in anti-mouse horseradish peroxidase (HRP)-labelled secondary antibody for 2 h (Sigma, UK). The 3,3-diaminobenzidine (DAB) (Sigma, UK) was used for band detection.

### Optimization methods and predictive modeling

#### Response surface methodology

After the initial expression of antiEpEX-scFv, we employed the RSM-CCD methodology for optimization of soluble expression of antiEpEX-scFv, using software package Design-Expert version 11 (Stat-Ease Inc., Minneapolis, USA). Based on our previously published data, the effects of independent variables including post-induction temperature, post-induction time, optical cell density in 600 nm before the induction and concentration of inducer as numerical factors and effect of different strains as a categorical factor on the production of soluble antiEpEX-scFv fragment were examined in the current study. Each numerical variable was set to 5 levels with 2 replications: plus and minus 1 (factorial points), plus and minus alpha (axial points), and the central point (12 central points and 48 non-central points in total) (Table 1). Then the categorical factor with 4 levels was added, a total of 232 separate experiments were carried out in 250 mL Erlenmeyer flasks containing 50 mL of TY2x medium (Supplementary Table 1). The estimated response obtained from RSM model was further compared with actual response in terms of coefficient of determination (R^2^) and Root mean square error (RMSE) using the Eqs. (1) and (2).

1 $${R}^{2}=1-\frac{{\sum }_{i=1}^{n}({{y}_{i}-{y}_{di)}}^{2}}{{\sum }_{i=1}^{n}({{y}_{di}-{y}_{a)}}^{2}}$$

2 $$RMSE=\sqrt{\frac{1}{n}{\sum }_{i=1}^{n}({{y}_{i}-{y}_{di)}}^{2}}$$

where *n* represents the number of experiments, *y*_*i*_, the predicted value, *y*_*di*_ the experimental value and *y*_*a*_ is the average of experimental value.

**Table 1:** Coded values of numerical and categorical variables used in central composite design.

Factors	Name	Type	Minimum	Maximum	Coded Low	Coded high	Central
A	Time (h)	Numeric	0	32	−1 ↔ 8.00	+ 1 ↔ 24.00	16
B	Temperature (°C)	Numeric	16	44	−1 ↔ 23.00	+ 1 ↔ 37.00	30
C	OD	Numeric	0.5	0.9	−1 ↔ 0.60	+ 1 ↔ 0.80	0.7
D	IPTG concentration (mM)	Numeric	0.2	1	−1 ↔ 0.40	+ 1 ↔ 0.80	0.6
E	Strain	Categoric	BW25113(DE3), Origami(DE3), SHuffle T7, BL21(DE3)				

#### Artificial neural network

**Figure 1:**
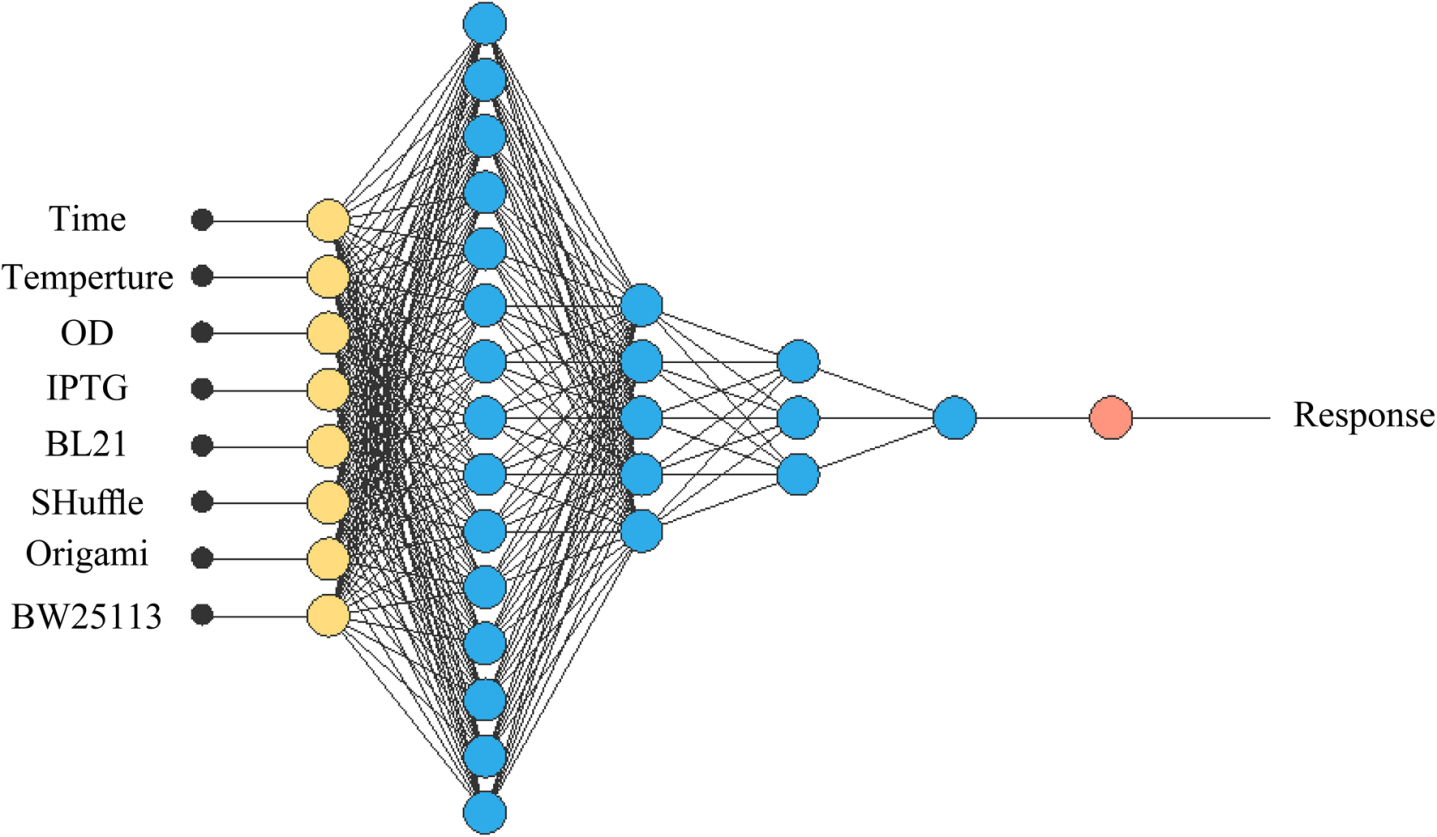
Multilayer feed forward neural network for eight input variables, 15, 10 and 3 neurons in the first, second and last hidden layers respectively and one output layer.

Alongside RSM methodology, we used ANN for optimization. In this study, Neural Designer software version 4.2.0 by Artelnics company feed-forward backpropagation in Multi-layer perceptron (MLP) was employed with 4 numerical and 1 categorical factor (with 4 levels). A multi-layer neural architecture contains input, output and hidden layers. The input layer consisting of eight neurons represents the variables including post-induction temperature, post-induction time, optical cell density in 600 posnm before the induction and concentration of inducer and four different strains (BW25113(DE3), Origami(DE3), SHuffle T7 and BL21(DE3)). The raw results of densitometry analysis were used for input (Supplementary Table 1). The output layer with one neuron represents soluble expression of antiEpEX-scFv (Fig. 1). The neurons number in the hidden layer was chosen depend on R^2^. Finally, considering R^2^ and RMSE, the estimated response obtained from ANN model was compared with actual response using the Eqs. (1) and (2).

### Comparison of predictive capabilities and validation of the RSM and ANN-based models

The prediction capabilities of RSM and ANN models were compared using error parameters. A dataset having 145 data points was randomly selected from the total dataset. The actual response of protein solubility was compared with estimated response achieved by RSM and ANN model in the randomly selected dataset in terms of R^2^ and RMSE using the Eqs. (1) and (2). Smaller values of RMSE show fair performance of the prediction models. Moreover, the experimental response of soluble protein production was plotted along with the corresponding predicted values of the RSM and ANN models. In addition, the validity of the models was evaluated by experimentally assessing the combination of tested variables leading to the maximum predicted level of protein solubility.

## Results

### Protein expression

**Figure 2:**
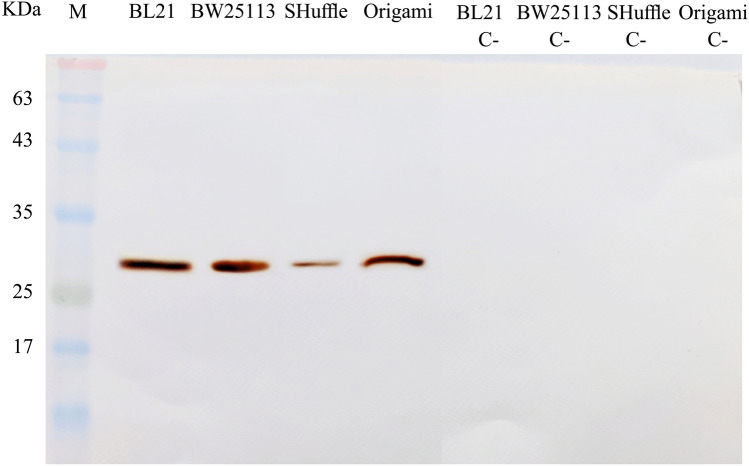
Western blotting analysis of the antiEpEX-scFv recombinant protein. Bacterial lysates of BL21(DE3), BW25113(DE3), SHuffle T7 and Origami(DE3) before (C-) and after induction were electrophoresed. After separation, protein bands were transferred onto the PVDF membrane and treated with anti-6 × His tag antibody.

The expression of the scFv protein was assessed in four *E. coli* strains before optimization using SDS-PAGE method. Utilizing western blotting, anti-His-tag monoclonal antibody can confirm the expression of His –tagged scFv in all stains studied here (Fig. 2).

### Predictive modeling and optimization methods

#### Response surface methodology modeling

Based on the published data, four numerical (post-induction time, concentration of inducer, post-induction temperature, and optical cell density) and one categorical (different strains) factors were selected for statistical optimization. As presented in Table 1, the five-level CCD with a total of 232 runs was employed (Supplementary Table 1). The dependent response (soluble production of scFv) was correlated with the independent numerical factors (coded values) in different strains using predicted following equations:

$$ \begin{aligned} & \left( {\text{Y}} \right)^{{0.5}} : \\ & {\text{BW }}25113\left( {{\text{DE}}}3 \right) \\ & {\text{Y}} = \, - 36.1443A + 137.399B - 6761.67C + 2608.58D \\ & \qquad- \, 0.501425AB + 17.572AC - 35.8366AD - 32.7466BC \\ & \qquad+ 48.2716BD - 2,588.53CD + 2.22388A^{2} - 2.01959B^{2} \\ & \qquad+ 7078.58C^{2} - 1294.25D^{2} + 420.482 \\ \end{aligned} $$

$$ \begin{aligned} & {\text{Origami}}\left( {{\text{DE}}} 3\right) \\ & {\text{Y}} = 209.604A - 40.9083B - 12140.1C - 91.7678D \\ &\qquad - 1.83904AB - 108.763AC - 16.7602AD + 167.013BC \\ & \qquad- 17.7474BD - 162.912CD - 1.43726A^{2} - \, 0.861316B^{2} \\ & \qquad+ 5869.2C^{2} + 447.055D^{2} + 4861.88 \\ \end{aligned} $$

$$ \begin{aligned} & {\text{SHuffle T}}7 \\ & 173.319A + 120.981B + 13795C + 1830.34D - 1.46395AB \\ &\qquad - 34.9288AC - 66.2975AD - 129.442BC - 7.38606BD \\ &\qquad - 4836.21CD - 2.36028A^{2} + \, 0.178132B^2 - 4977.67C^{2} \\ & \qquad+ 1802.92D^{2} - 7034.69 \\ \end{aligned} $$

$$ \begin{aligned} & {\text{BL}}21\left( {{\text{DE}}} 3\right) \\ &\qquad - 9.03435A - 2.64382B - 7902.75C - 687.916D + 1.13725AB \\ &\qquad - 134.612AC + 37.2579AD + 45.6254BC + 85.7176BD \\ &\qquad - 5129.02CD + 1.81334A^{2} - 1.73011B^{2} + 8359.54C^{2} \\ &\qquad + 853.879D^{2} +4084.09 \\ \end{aligned} $$

In the above equations, Y denotes response (soluble production of anti EpEX-scFv), and A, B, C, and D denotes post-induction time, post-induction temperature, cell density before induction, and IPTG concentration, respectively.

According to ANOVA results, significant "F value" (15.78) as well as insignificant "Lack of Fit for value of F" indicates that the model is valid to predict soluble production of scFv. The low *p*-value (Prob > F) (< 0.0001) of the model resignifies its significance. R^2^ (the coefficient of determination) of 0.950 implies that 95.0% of the variability in the response can be described by the model. Furthermore, the difference value less than 0.2 confirms a high degree of correlation between the predicted R^2^ (0.7487) and adjusted R^2^ (0.7906) values. Plot illustrated in Supplementary Fig. S1 confirms this correlation again. Also, the accuracy and predictability of the selected model were validated by the normal probability plot of the studentized residuals (Supplementary Fig. S1). Based on ANOVA results, the proposed model fits the experimental data well. So it can be effectively utilized to navigate the design space (Table 2).

**Table 2:** Analysis of variance for the experimental results of the central-composite design for soluble production of anti EpEX-scFv.

Source	Sum of Squares	df	Mean Square	F-value	*P*-value
Model	1.697E + 07	59	2.876E + 05	15.78	< 0.0001
A-time	7.430E + 05	1	7.430E + 05	40.78	< 0.0001
B-temp	27,256.37	1	27,256.37	1.50	0.2230
C-OD	22,835.55	1	22,835.55	1.25	0.2645
D-IPTG	5.929E + 05	1	5.929E + 05	32.54	< 0.0001
E-strain	1.332E + 06	3	4.439E + 05	24.36	< 0.0001
AB	1.785E + 05	1	1.785E + 05	9.80	0.0021
AC	3.481E + 05	1	3.481E + 05	19.10	< 0.0001
AD	1.365E + 05	1	1.365E + 05	7.49	0.0069
AE	2.131E + 06	3	7.103E + 05	38.99	< 0.0001
BC	9977.13	1	9977.13	0.5476	0.4603
BD	1.858E + 05	1	1.858E + 05	10.20	0.0017
BE	1.500E + 06	3	4.998E + 05	27.43	< 0.0001
CD	5.175E + 05	1	5.175E + 05	28.40	< 0.0001
CE	7.618E + 05	3	2.539E + 05	13.94	< 0.0001
DE	7.439E + 05	3	2.480E + 05	13.61	< 0.0001
A^2^	1882.41	1	1882.41	0.1033	0.7483
B^2^	6.291E + 05	1	6.291E + 05	34.53	< 0.0001
C^2^	3.555E + 05	1	3.555E + 05	19.51	< 0.0001
D^2^	69,859.91	1	69,859.91	3.83	0.0518
ABE	5.310E + 05	3	1.770E + 05	9.72	< 0.0001
ACE	2.966E + 05	3	98,873.10	5.43	0.0014
ADE	4.655E + 05	3	1.552E + 05	8.52	< 0.0001
BCE	7.396E + 05	3	2.465E + 05	13.53	< 0.0001
BDE	4.444E + 05	3	1.481E + 05	8.13	< 0.0001
CDE	2.047E + 05	3	68,242.47	3.75	0.0122
A^2^E	2.078E + 06	3	6.928E + 05	38.02	< 0.0001
B^2^E	3.756E + 05	3	1.252E + 05	6.87	0.0002
C^2^E	6.003E + 05	3	2.001E + 05	10.98	< 0.0001
D^2^E	4.297E + 05	3	1.432E + 05	7.86	< 0.0001
**Residual**	3.134E + 06	172	18,219.75		
Lack of Fit	6.933E + 05	32	21,666.07	1.24	0.1956
Pure Error	2.440E + 06	140	17,432.02		
**Cor Total**	2.010E + 07	231			

As depicted in Table 2, three linear terms (post-induction time (A), concentration of inducer (D) and different strains (E)) were found to be significant for soluble production of scFv whereas post-induction temperature and optical cell density variables had no significant impact on solubility of scFv. All interactive terms except temperature- optical cell density (BC) were found to be significant which was evident from their *p*-values (less than 0.05). Also, two quadratic terms (A^2^ and D^2^) were not significant according to Table 2. Moreover, it can be concluded that post-induction time is largely affecting soluble production of anti EpEX-scFv.

**Figure 3:**
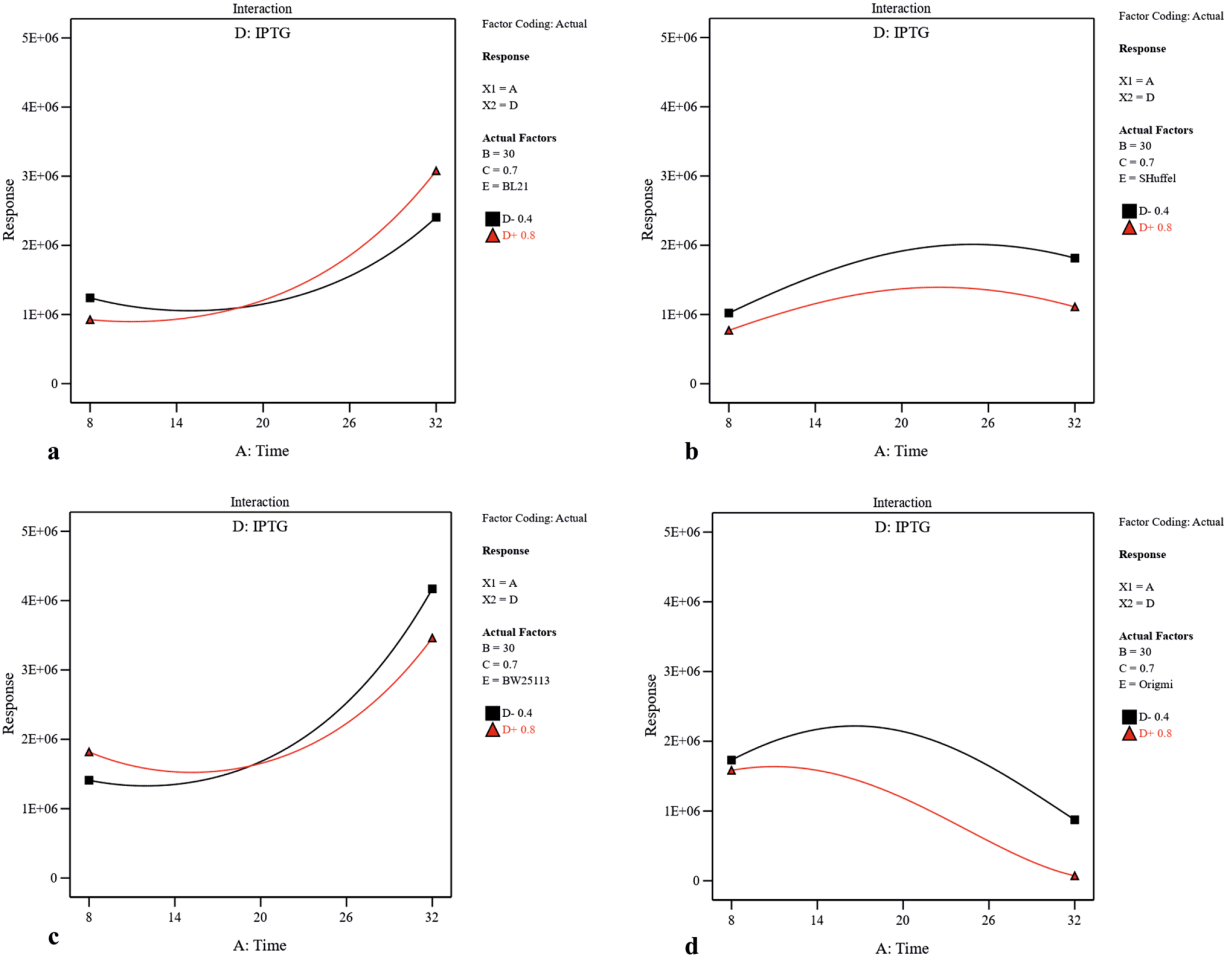
The interactive effects of post-induction time and inducer concentration on soluble production of scFv in (**a**) BL21(DE3), (**b**) SHuffle T7, (**c**) BW25113(DE3), and (**d**) Origami(DE3). Post-induction temperature (B = 30 °C) and cell density of induction time (C = 0.7) were kept at their constant middle levels.

**Figure 4:**
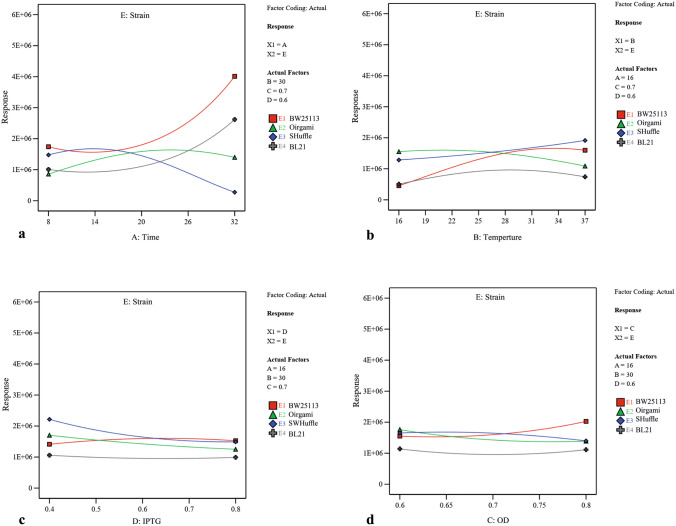
The interactive effects between strain type and (**a**) post-induction time (**b**) post-induction temperature (**c**) inducer concentration and (**d**) cell density of induction time. In each case other three numerical factors were kept at their constant middle levels (post-induction time (A), post-induction temperature (B), cell density of induction time (C), and inducer concentration (D).

Utilizing two-dimensional graphs, the interactive effects between two significant independent variables (A and D (Fig. 3), A and B (Supplementary Fig. S2), A and C (Supplementary Fig. S3), B and D (Supplementary Fig. S4) and C and D (Supplementary Fig. S5)) were studied in different strains while keeping other two numerical factors at their constant middle levels. From Fig. 3, and Supplementary Fig. S2 and S3, it was evident that increasing the post-induction time led to solubility increase in three strains including BW25113(DE3), Origami(DE3) and BL21(DE3), and decrease in SHuffle T7. Moreover, upon increasing the concentration of inducer, the solubility had significantly decreased in Origami(DE3) and SHuffle T7 which was more substantial in SHuffle T7 than that in Origami(DE3) in similar post-induction time (Fig. 3). Also, increasing the temperature had a negative effect on scFv solubility in Origami(DE3) (Supplementary Fig. S2). As illustrated in Supplementary Fig. S3, more soluble protein was provided in BW25113(DE3) when protein production was induced at higher OD600 nm while the amount of soluble scFv obtained in Origami(DE3) and SHuffle T7 had been negatively affected by increasing the OD600 nm before induction. A significant interaction between temperature and inducer concentration is also indicated by ANOVA (*p*-value of 0.0017) (Table2). As depicted in Supplementary Fig. S4, when the levels of post-induction time (A) and optical cell density (C) were kept constant at their medium value (16 and 0.7 respectively), temperature raise could lead to increase the solubility in BW25113(DE3) and SHuffle T7. In BW25113(DE3), although increasing IPTG concentration at lower temperature decreased the amount of soluble fraction, an increase in inducer concentration at higher temperature had a positive effect on protein solubility. The dependency of OD600 nm before induction (C) and IPTG concentration (D) on scFv solubility when the post-induction time (A) as well as temperature (B) is kept constant (16 °C and 30 °C respectively) is illustrated in Supplementary Fig. S5. According to this graph, an increase in OD600 nm at higher IPTG concentration (0.8) led to a decrease in solubility in BL21(DE3) and SHuffle T7 and at lower inducer concentration (0.4), increasing the OD600 nm enhanced protein solubility. Interestingly, Supplementary Fig. S5 also declares that increasing the OD600 nm at both IPTG concentration levels leads to a solubility increase in BW25113(DE3) and decrease in Origami(DE3). The interactive effects between each independent numerical variable and strain type were studied while keeping other three numerical factors at their constant middle levels. As depicted in Fig. 4 and confirmed by ANOVA results, post-induction time was the most effective factor on soluble production of scFv in four strains studied here.

#### Artificial neural network modeling

Using artificial neural network (ANN) models, the behavior of nonlinear multivariate systems can be predicted. The multilayer feed forward neural network with Quasi-Newton algorithm was the model considered for the present work. In this study, the same DoE used in building the RSM model was also employed to develop the ANN-based model. The experimental data was divided into three subsets including training, testing and validation (70%, 15%, 15% of data respectively) (Table 3). A small amount of noise was added to the data set and regularization of weight was done to prohibit overfitting the training data and make smoother responses. The network topology developed for ANN determines the accuracy of a model prediction. To achieve optimal ANN structure for prediction, the number of hidden layers and neural composition were determined by varying the number of hidden layers (1–5) as well as number of neurons (8–48). We had 8 neurons in the input layer and the scaling layers were set at automatic with 8 neurons. For perceptron layers, different architectures were investigated and best results were achieved when we had 15, 10 and 3 neurons in the first, second and last hidden layers respectively. Activation function in all hidden layers was a hyperbolic tangent. The scaled outputs from the hidden layers connected to the unscaled layer with one neuron to produce the original units. Moreover, the model selection was carried out to achieve better network architecture with the best generalization. Finally, the performance of the developed network was examined based on NRMSE and R^2^ of testing data. The fitness of the model was confirmed by its overall R^2^ which was found to be 0.87. NRMSE value also indicates a good prediction of outputs (0.288).

**Table 3:** The number and percentage of experimental data used for training, testing and validation in artificial neural network.

The number of experimental data	The percentage of experimental data	
164	70.6%	Train
34	14.7%	Test
34	14.7%	Validation
232	100%	Total

### Comparison of predictive capabilities and validation of the RSM and ANN-based models

**Figure 5:**
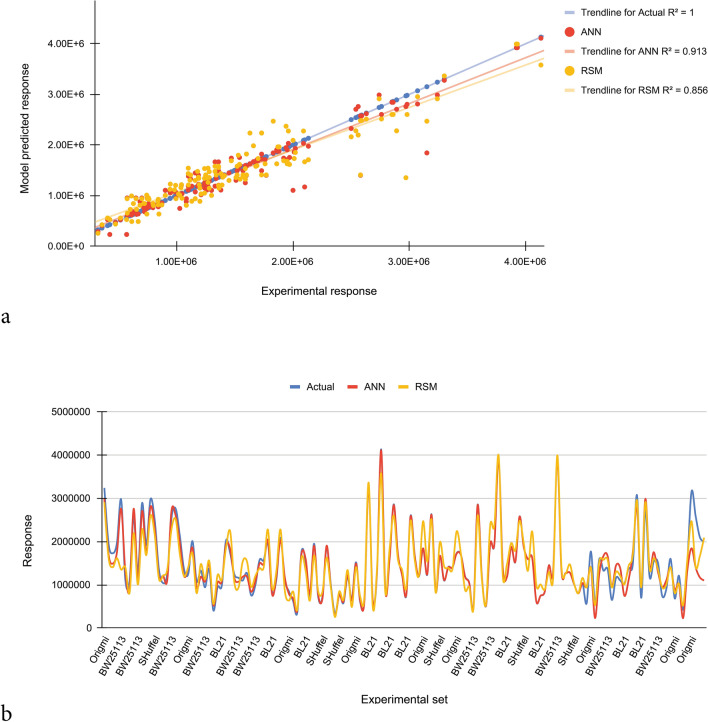
Comparison of prediction capabilities of RSM and ANN models for randomly selected dataset. (**a**) RSM and ANN predicted vs. actual responses. (**b**) Comparison of responses obtained from experimental, RSM and ANN. The ANN model has fitted the experimental responses with an excellent accuracy. Greater deviation is seen in RSM-based prediction for soluble production of scFv than ANN.

In the current study, based on R^2^ and the error analyses, the effectiveness of the empirical models was statistically evaluated between estimated and actual responses. A dataset having 145 data points was randomly selected from the total dataset. The experimental response along with the predicted data obtained for soluble production of scFv are given in Supplementary Table 2. According to obtained results, for random dataset, the R^2^ for ANN and RSM models are 0.913 and 0.856 respectively, demonstrating the ability of these models to describe 91% and 85% of the variations of the actual values respectively. The NRMSE is more for RSM model (0.264) than for the ANN model (0.154), which means that the predicting capacity of the ANN model is higher over the RSM model. According to comparative plot for predicted and actual values, the ANN model has fitted the experimental responses with an excellent accuracy. Greater deviation is seen in RSM-based prediction for soluble scFv yield than ANN (Fig. 5). For validation of models, utilizing the RSM model based predicted optimum conditions (Table 4), experimental densitometric analysis result of 112.4 mg/L was obtained for soluble fraction which was in good correlation with the predicted value of 97.9 mg/L. When the levels of the variables were replaced in the ANN model, the maximum predicted response value was 106.1 mg/L, which was closer to the experimental result (112.4 mg/L) than the RSM (97.9 mg/L). Reaffirms the higher accuracy of ANN model.

**Table 4:** Optimum condition and strain for soluble production of anti EpEX-scFv.

Input variables	RSM model	ANN model
Time	24 h	24 h
Temperature	23 °C	23 °C
Optical density	0.6	0.6
Inducer concentration	0.4 mg/ml	0.4 mg/ml
Strain	Origami(DE3)	Origami(DE3)
Experimental data	112.4 mg/L	112.4 mg/L
Predicted data	97.9 mg/L	106.1 mg/L

## Discussion

Due to unsuitable folding of protein, most of the heterologous proteins expressed in *E. coli* aggregate in inclusion bodies which are partly or completely devoid of biological activity. Solubilization of these aggregates requires denaturing agents in a high concentration causing the loss of secondary structure of protein. Moreover, after refolding, the obtained proteins might be unstable. Therefore, focusing on environmental modification, many investigations have tried to develop expression of well folded highly soluble proteins in *E. coli* during the past three decades^13^.

The RSM and ANN models for optimized soluble production of scFv were studied here for the first time. Also, here, the effects of four numerical factors along with different strains as a categorical factor on response were investigated for the first time. Based on the developed quadratic model, temperature and induction OD were not significant and the other three terms (post-induction time, concentration of inducer and different strains) were found to be significant for soluble production of scFv. In agreement with our study, many investigations showed the influence of different *E. coli* strains on solubility of various proteins. For example, the effect of different engineered hosts including BL21(DE3) pLysS, BL21(DE3) and Rosetta on soluble expression of recombinant TNF-α was assessed by papaneophytou et al. Their results showed lower yield of soluble TNF-α in Rosetta compared to the other two hosts^14^. The effective role of the engineered strains on solubility demonstrated here is also in line with Zhang et al.'s study which has showed the higher solubility of IGF1-thioredoxin fusion in Rosetta-gami (DE3) than that in Rosetta (DE3) and Bl21 (DE3) bacteria^15^. Herein, the maximum soluble amount of scFv was achieved in *E. coli* Origami(DE3) which is a type of mutant strain with mutation in thioredoxin reductase (trxB) and glutathione reductase (gor) genes. Its oxidative environment enhances disulfide bonds formation in the cytoplasm which leads to lesser accumulation of misfolded proteins and inactive inclusion body formation^16^. Moreover, the optimal culture conditions obtained here were IPTG concentration of 0.4 mM, cell density before induction of 0.6 nm, post-induction temperature of 23 °C and post-induction time of 24 h. Consistent with this finding, Heo et al. achieved the highest soluble amount of anti-c Met scFv in 0.5 mM concentration of IPTG. They showed that in Origami (DE3), higher inclusion body formation was associated with higher concentrations of IPTG and lowering IPTG concentration (1 to 0.5 mM) led to higher levels of functional anti-c Met scFv expression. This is because lesser inducer concentration can lead to lower transcription rate and higher efficiency of intracellular folding of the target protein^16^. Consistently, soluble production of recombinant scFv against HBV preS2 in Origami2 (λDE3) was shown to be promoted at low concentration of inducer^17^. We also showed that maximum soluble amount could be achieved at low temperature (23 °C). Our data was in agreement with the prior studies in which several proteins including human interferon α-2 ricin A chain, subtilisin E, Fab fragments, and β-lactamase had higher solubility at low temperatures^18,19^. Similarly, Emamipour et al. achieved maximum solubility at 23 °C for DsbA-IGF1 protein using BBD methodology^20^. This may be a result of providing enough time for the proper folding due to slow rate of cell processes such as transcription, translation, and cell division. Also, decreasing temperature has been shown to eliminate the heat-shock proteases which are induced during overexpression of heterologous proteins. Also, it has been reported that at low temperature, the expression and activity of some chaperones are increased which can facilitate corrected folding of the recombinant proteins^21^. The results of the current study also showed that a long incubation time was critical for the optimal expression of soluble scFv. This finding was consistent with the findings of Sina et al. which showed a significant increase in soluble expression of humanized anti-TNF-α scFv- GST fusion protein in *E. coli* Origami (DE3) when it was produced in the presence of low amount of inducer, in low cultivation temperature under a long incubation time^22^.

In the current study, RSM and ANN methodologies are compared for their efficiency in optimization of fermentation media. Although both methods were shown to be effective in determining the optimum conditions to improve the response, comparing R^2^ achieved from ANN model (0.913) to that obtained for RSM (0.856) showed the better ability of the former in modeling soluble production of scFv, due to its deliberate overtraining. Consistently, for culture medium optimization, machine learning techniques have been shown to outperform the statistically-designed models in few investigations presented in the literature. For example, to maximize growth and lipid productivity of marine microalga *Tetraselmis sp*, the composition of a culture medium was optimized by Mohamed et al. using both RSM and ANN models. They reported that ANN was a more appropriate method for increasing biomass concentration and lipid yield than the RSM-based optimization method^23^. Similarly, compared to RSM, a higher predictive capacity for ANN was reported by Rafigh et al. for optimizing the culture conditions for curdlan production by *Paenibacillus polymyxa*^24^.

## Conclusion

In the present study, we have optimized fermentation condition for soluble production of antiEpEX-scFv by optimizing four literature based numerical factors and one categorical variable using ANN and RSM. Based on the RSM, three linear terms (post-induction time (A), concentration of inducer (D) and different strains (E)) significantly affected solubility of scFv whereas post-induction temperature and optical cell density variables had no significant impact on the response. Moreover, post-induction time was the most affecting parameter. Analysis of error parameters and R^2^ from a dataset having 145 data points randomly selected from the total dataset revealed the superiority of ANN model to RSM. Thus it may be concluded that although RSM usually is the first choice for statistical modelling, machine learning models can also be utilized to optimize the fermentation condition. The best fermentation conditions estimated by RSM, (induction at cell density 0.6 with 0.4 mM IPTG for 24 h at 23 °C in Origami(DE3)), allowed predicting a maximum soluble production of 97.9 mg/L which was in good correlation with the experimental value of 112.4 mg/L However, predicted value by ANN model (106.1 mg/L) was closer to the experimental result (112.4 mg/L) than that predicted by RSM (97.9 mg/L). Encouraging results of this study show that machine learning approaches can be applied for efficient soluble production of scFv which is highly applicable in diagnostic and therapeutic purposes.

## Supplementary Information

Supplementary Information 1.

Supplementary Information 2.

Supplementary Information 3.

Supplementary Information 4.

Supplementary Information 5.

Supplementary Information 6.

Supplementary Information 7.
