# Supplementary material for: Machine learning modeling for solubility prediction of recombinant antibody fragment in four different E. coli strains
Source: Sci Rep. 2022 Mar 31;12:5463. doi: 10.1038/s41598-022-09500-6 (PMC8971470; doi:10.1038/s41598-022-09500-6)
Supplement: Supplementary file 1 — Supplementary Information 1. [file 41598_2022_9500_MOESM1_ESM.doc]

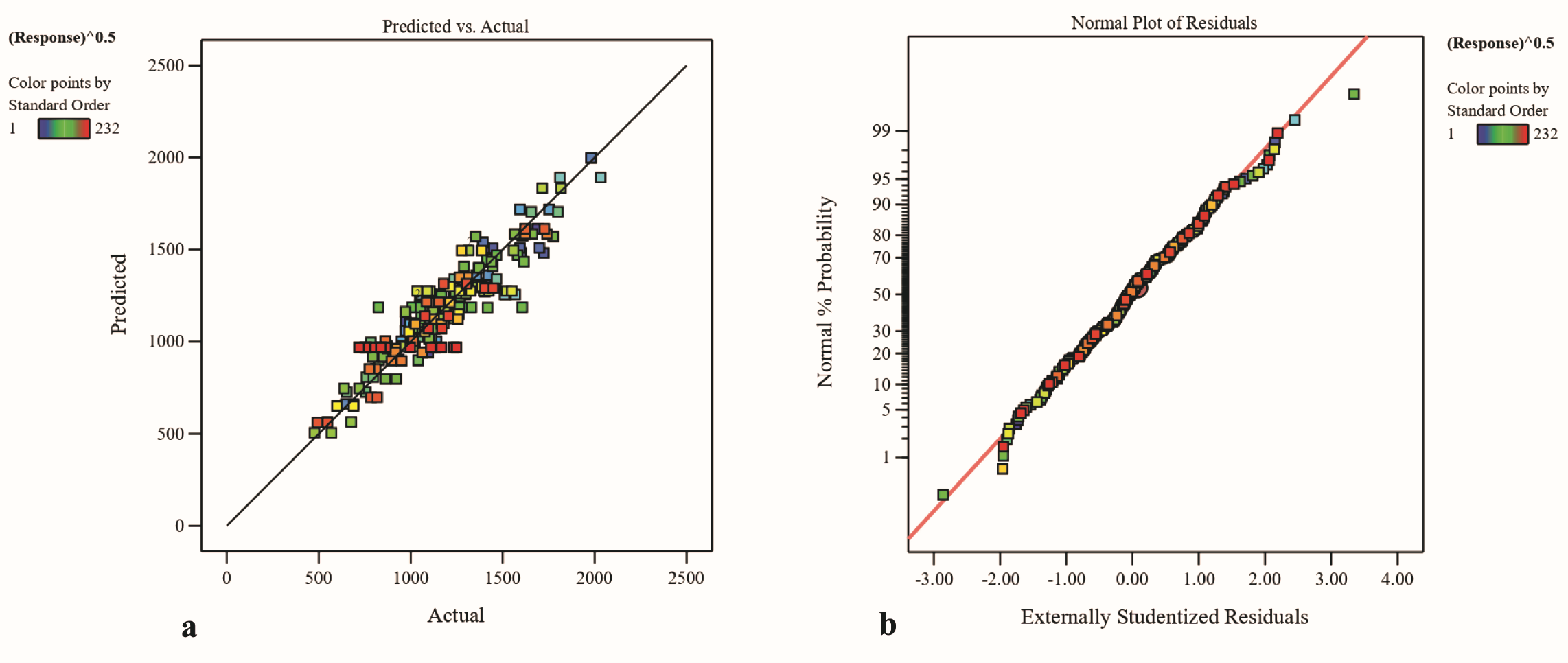


**Figure S1.** Regression model adequacy diagnostic plots for soluble production of antiEpEX-ScFv. a) predicted vs actual plots and b) normal plot.
