# Supplementary material for: Machine learning modeling for solubility prediction of recombinant antibody fragment in four different E. coli strains
Source: Sci Rep. 2022 Mar 31;12:5463. doi: 10.1038/s41598-022-09500-6 (PMC8971470; doi:10.1038/s41598-022-09500-6)
Supplement: Supplementary file 3 — Supplementary Information 3. [file 41598_2022_9500_MOESM3_ESM.doc]

**
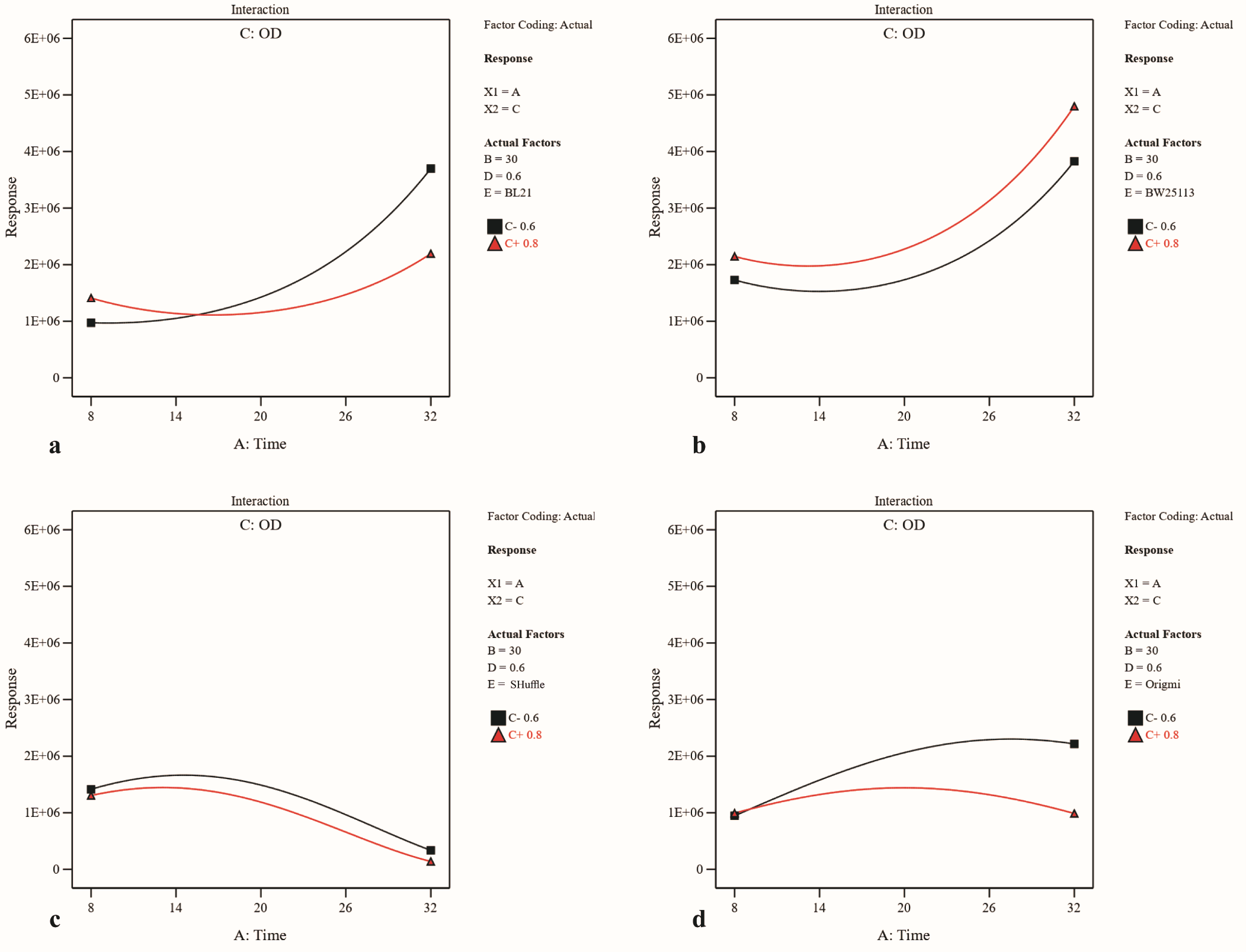
**

**Figure S3.** The interactive effects of post-induction time and cell density of induction time on soluble production of scFv in a) BL21 (DE3), b) BW25113 (DE3), c) SHuffle T7 and d) Origami (DE3). Post-induction temperature (B= 30 °C) and inducer concentration (D= 0.6) were kept at their constant middle levels.
