# Supplementary material for: Machine learning modeling for solubility prediction of recombinant antibody fragment in four different E. coli strains
Source: Sci Rep. 2022 Mar 31;12:5463. doi: 10.1038/s41598-022-09500-6 (PMC8971470; doi:10.1038/s41598-022-09500-6)
Supplement: Supplementary file 4 — Supplementary Information 4. [file 41598_2022_9500_MOESM4_ESM.doc]

**
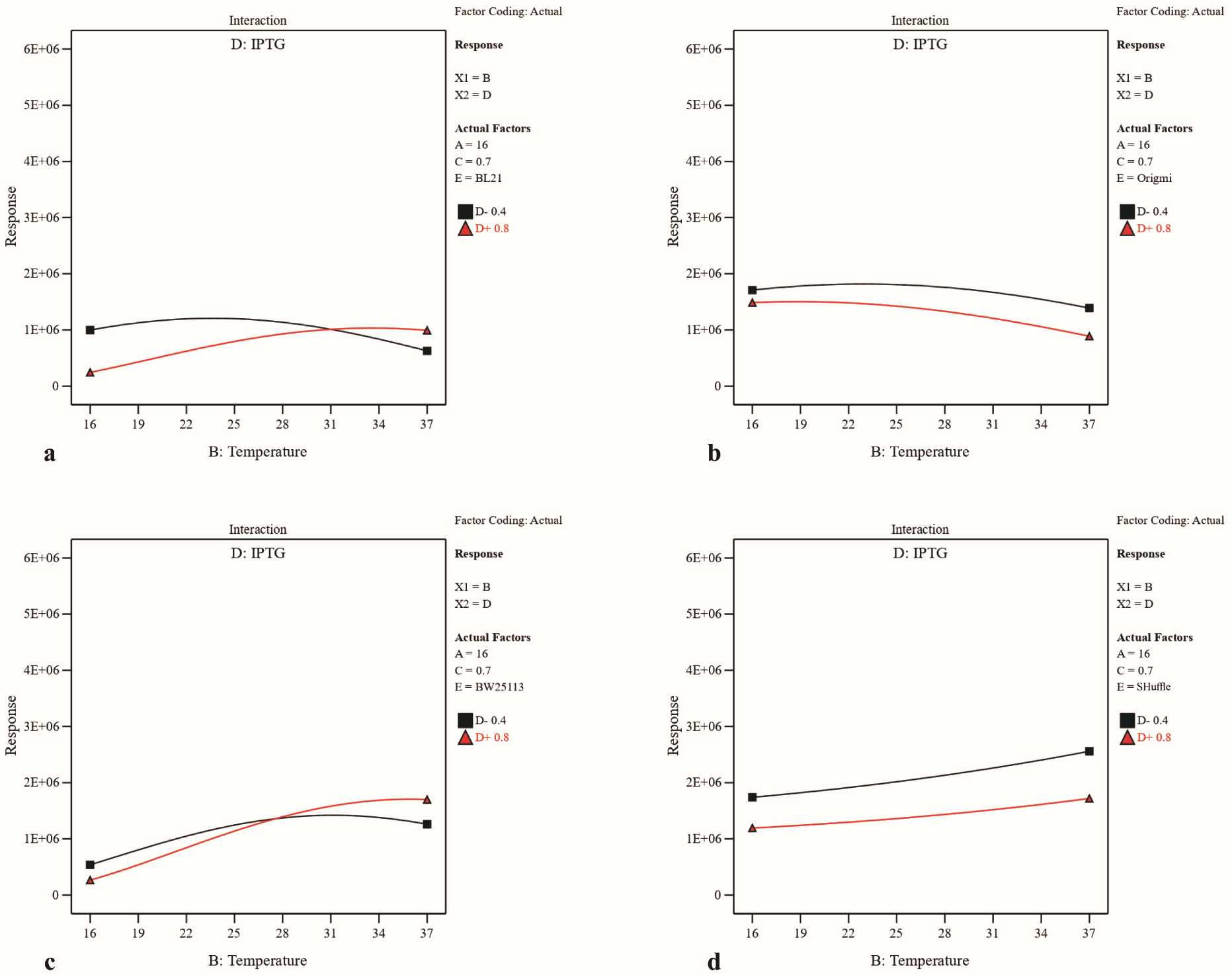
**

**Figure S4.** The interactive effects of post-induction temperature and inducer concentration on soluble production of scFv in a) BL21 (DE3), b) Origami (DE3), c) BW25113 (DE3), and d) SHuffle T7. Post-induction time (A= 16 h) and cell density of induction time (C= 0.7) were kept at their constant middle levels.
