# Supplementary material for: Machine learning modeling for solubility prediction of recombinant antibody fragment in four different E. coli strains
Source: Sci Rep. 2022 Mar 31;12:5463. doi: 10.1038/s41598-022-09500-6 (PMC8971470; doi:10.1038/s41598-022-09500-6)
Supplement: Supplementary file 5 — Supplementary Information 5. [file 41598_2022_9500_MOESM5_ESM.doc]

**
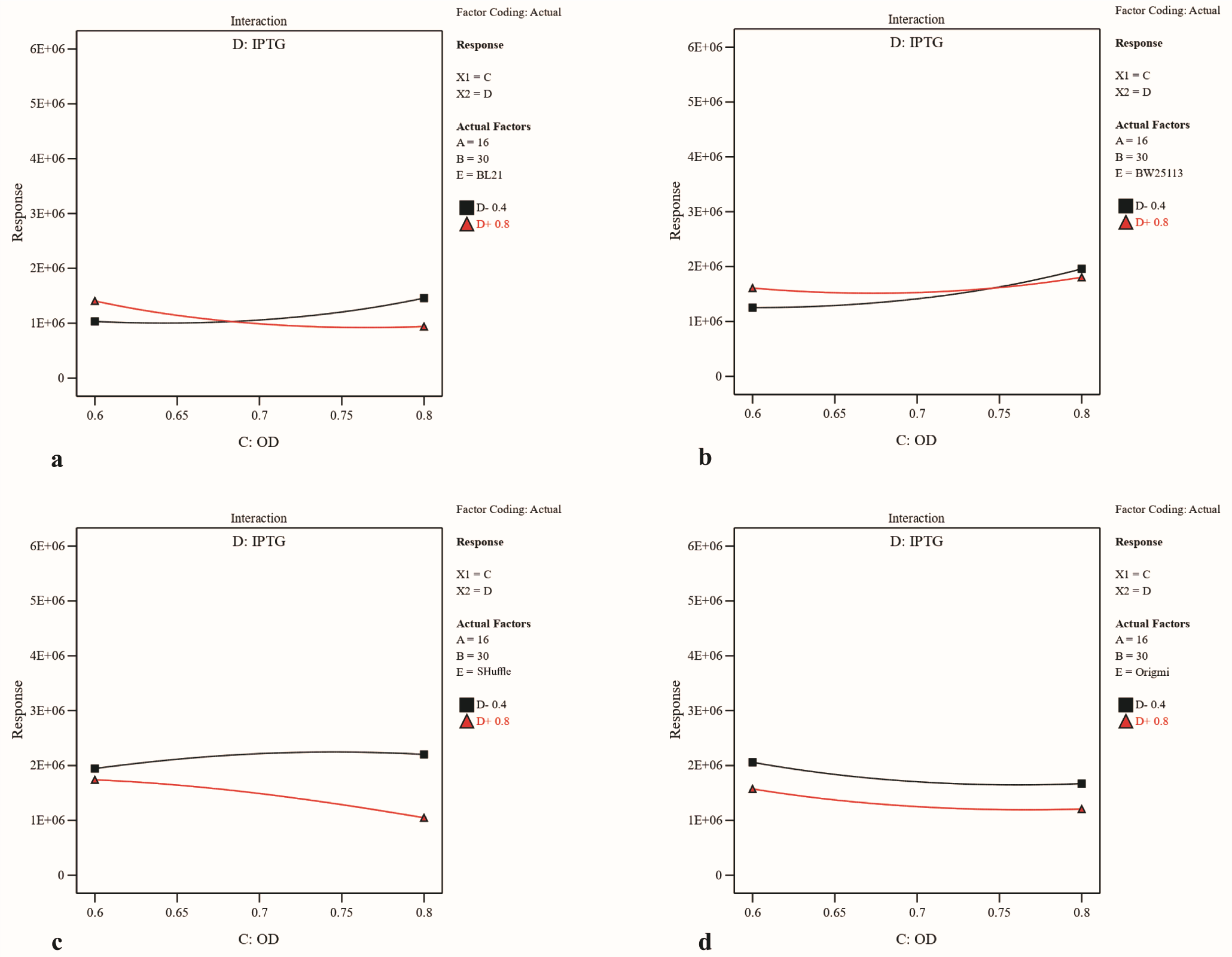
**

**Figure S5.** The interactive effects of cell density of induction time and inducer concentration on soluble production of scFv in a) BL21 (DE3), b) BW25113 (DE3), c) SHuffle T7, and d) Origami (DE3). Post-induction time (A= 16 h) and post-induction temperature (B= 30 °C) were kept at their constant middle levels.
