# Supplementary material for: Machine learning modeling for solubility prediction of recombinant antibody fragment in four different E. coli strains
Source: Sci Rep. 2022 Mar 31;12:5463. doi: 10.1038/s41598-022-09500-6 (PMC8971470; doi:10.1038/s41598-022-09500-6)
Supplement: Supplementary file 6 — Supplementary Information 6. [file 41598_2022_9500_MOESM6_ESM.doc]

Supplementary Table S1. Central composite design of numerical and categorical variables and their corresponding actual and RSM and ANN predicted values.

| **Run number** | **Time (h)** | **Temperature (°C)** | **OD600 (nm)** | **IPTG** | **Strain** | **RSM** | **Actual** | **ANN** |
| --- | --- | --- | --- | --- | --- | --- | --- | --- |
| 1 | 8 | 23 | 0.6 | 0.8 | BW25113 | 1.15E+06 | 1.14E+06 | 1148819.549 |
| 11 | 8 | 23 | 0.8 | 0.8 | BW25113 | 1.28E+06 | 1.30E+06 | 1356782.336 |
| 12 | 24 | 37 | 0.6 | 0.4 | BW25113 | 1.66E+06 | 1.75E+06 | 1717384.04 |
| 13 | 24 | 37 | 0.8 | 0.8 | BW25113 | 1.94E+06 | 1.95E+06 | 2369598.423 |
| 16 | 16 | 30 | 0.7 | 0.6 | BW25113 | 1.23E+06 | 1.67E+06 | 1578767.12 |
| 17 | 16 | 30 | 0.7 | 0.6 | BW25113 | 1.33E+06 | 1.67E+06 | 1578767.12 |
| 21 | 16 | 16 | 0.7 | 0.6 | BW25113 | 474699 | 467348.532 | 437291.2384 |
| 26 | 16 | 30 | 0.7 | 0.6 | BW25113 | 1.36E+06 | 1.67E+06 | 1578767.12 |
| 29 | 24 | 37 | 0.6 | 0.4 | BW25113 | 1.65E+06 | 1.75E+06 | 1717384.04 |
| 30 | 16 | 30 | 0.7 | 0.6 | BW25113 | 1.12E+06 | 1.67E+06 | 1578767.12 |
| 32 | 16 | 30 | 0.5 | 0.6 | BW25113 | 2.02E+06 | 1.93E+06 | 1851885.506 |
| 33 | 8 | 37 | 0.6 | 0.8 | BW25113 | 1.96E+06 | 2.03E+06 | 2275180.057 |
| 44 | 8 | 37 | 0.6 | 0.8 | BW25113 | 2.09E+06 | 2.03E+06 | 2275180.057 |
| 46 | 24 | 23 | 0.8 | 0.8 | BW25113 | 1.83E+06 | 1.98E+06 | 1707778.512 |
| 51 | 8 | 23 | 0.8 | 0.8 | BW25113 | 1.51E+06 | 1.30E+06 | 1356782.336 |
| 55 | 8 | 37 | 0.8 | 0.4 | BW25113 | 1.71E+06 | 1.86E+06 | 1731987.603 |
| 56 | 16 | 30 | 0.7 | 0.6 | BW25113 | 1.68E+06 | 1.67E+06 | 1578767.12 |
| 57 | 8 | 37 | 0.8 | 0.8 | BW25113 | 2.89E+06 | 2.70E+06 | 2279073.316 |
| 58 | 16 | 30 | 0.7 | 0.6 | BW25113 | 1.23E+06 | 1.67E+06 | 1578767.12 |
| 61 | 16 | 30 | 0.5 | 0.6 | BW25113 | 1.84E+06 | 1.93E+06 | 1851885.506 |
| 63 | 16 | 30 | 0.7 | 0.6 | BW25113 | 2.28E+06 | 1.67E+06 | 1578767.12 |
| 67 | 8 | 23 | 0.6 | 0.8 | BW25113 | 1.12E+06 | 1.67E+06 | 1148819.549 |
| 68 | 32 | 30 | 0.7 | 0.6 | BW25113 | 3.92E+06 | 3.92E+06 | 3992883.168 |
| 71 | 24 | 37 | 0.6 | 0.8 | BW25113 | 2.56E+06 | 2.76E+06 | 2195790.512 |
| 78 | 16 | 30 | 0.7 | 0.6 | BW25113 | 1.16E+06 | 1.67E+06 | 1578767.12 |
| 79 | 24 | 23 | 0.8 | 0.4 | BW25113 | 2.86E+06 | 2.84E+06 | 2601414.152 |
| 83 | 16 | 30 | 0.7 | 0.2 | BW25113 | 1.30E+06 | 1.10E+06 | 1004204.41 |
| 85 | 16 | 30 | 0.7 | 1 | BW25113 | 9.91E+05 | 1072103.07 | 1202816.693 |
| 86 | 24 | 23 | 0.8 | 0.4 | BW25113 | 2.85E+06 | 2.84E+06 | 2601414.152 |
| 87 | 8 | 37 | 0.6 | 0.4 | BW25113 | 9.45E+05 | 1053263.75 | 1226954.982 |
| 90 | 16 | 30 | 0.7 | 0.6 | BW25113 | 1.56E+06 | 1.67E+06 | 1578767.12 |
| 91 | 24 | 23 | 0.6 | 0.8 | BW25113 | 1.19E+06 | 1.22E+06 | 1340060.912 |
| 94 | 16 | 30 | 0.9 | 0.6 | BW25113 | 2.54E+06 | 2.81E+06 | 2953001.665 |
| 101 | 32 | 30 | 0.7 | 0.6 | BW25113 | 3.93E+06 | 3.92E+06 | 3992883.168 |
| 106 | 16 | 30 | 0.7 | 0.6 | BW25113 | 1.60E+06 | 1.67E+06 | 1578767.12 |
| 111 | 8 | 23 | 0.6 | 0.4 | BW25113 | 1.13E+06 | 1.54E+06 | 886346.9316 |
| 117 | 24 | 37 | 0.8 | 0.4 | BW25113 | 2.59E+06 | 2.58E+06 | 2480908.508 |
| 120 | 16 | 44 | 0.7 | 0.6 | BW25113 | 1.21E+06 | 1.08E+06 | 1123663.601 |
| 124 | 24 | 37 | 0.8 | 0.4 | BW25113 | 2.58E+06 | 2.58E+06 | 2480908.508 |
| 130 | 8 | 37 | 0.8 | 0.8 | BW25113 | 2.54E+06 | 2.70E+06 | 2279073.316 |
| 135 | 24 | 23 | 0.6 | 0.4 | BW25113 | 1.52E+06 | 1.33E+06 | 1579018.428 |
| 142 | 16 | 30 | 0.7 | 1 | BW25113 | 1.16E+06 | 1.07E+06 | 938961 |
| 145 | 16 | 30 | 0.7 | 0.6 | BW25113 | 2.46E+06 | 1.67E+06 | 1578767.12 |
| 151 | 24 | 23 | 0.6 | 0.4 | BW25113 | 1.12E+06 | 1.33E+06 | 1405196.868 |
| 153 | 8 | 37 | 0.8 | 0.4 | BW25113 | 2.01E+06 | 1.86E+06 | 1731987.603 |
| 157 | 24 | 37 | 0.8 | 0.8 | BW25113 | 1.94E+06 | 1.95E+06 | 2369598.423 |
| 164 | 8 | 37 | 0.6 | 0.4 | BW25113 | 1.20E+06 | 1.05E+06 | 1226954.982 |
| 173 | 16 | 44 | 0.7 | 0.6 | BW25113 | 9.43E+05 | 1077623.9 | 1123663.601 |
| 174 | 16 | 30 | 0.9 | 0.6 | BW25113 | 3.07E+06 | 2.81E+06 | 2953001.665 |
| 175 | 16 | 16 | 0.7 | 0.6 | BW25113 | 419590 | 467348.532 | 437291.2384 |
| 176 | 24 | 23 | 0.6 | 0.8 | BW25113 | 1.24E+06 | 1.22E+06 | 1340060.912 |
| 179 | 16 | 30 | 0.7 | 0.6 | BW25113 | 2.30E+06 | 1.67E+06 | 1578767.12 |
| 183 | 24 | 37 | 0.6 | 0.8 | BW25113 | 2.97E+06 | 2.76E+06 | 1352452.703 |
| 211 | 8 | 23 | 0.8 | 0.4 | BW25113 | 1.10E+06 | 1.19E+06 | 1541396.741 |
| 217 | 8 | 23 | 0.6 | 0.4 | BW25113 | 1.20E+06 | 1.19E+06 | 886346.9316 |
| 223 | 24 | 23 | 0.8 | 0.8 | BW25113 | 2.13E+06 | 1.98E+06 | 1707778.512 |
| 224 | 16 | 30 | 0.7 | 0.2 | BW25113 | 9.03E+05 | 1103672.92 | 1004204.41 |
| 229 | 8 | 23 | 0.8 | 0.4 | BW25113 | 1.27E+06 | 1.19E+06 | 1541396.741 |
| 2 | 24 | 37 | 0.8 | 0.8 | Origami | 1.08E+06 | 8.81E+05 | 809154.2209 |
| 4 | 16 | 30 | 0.7 | 1 | Origami | 1.28E+06 | 2.02E+06 | 1144985.602 |
| 8 | 16 | 30 | 0.7 | 0.2 | Origami | 2.00E+06 | 1.10E+06 | 2090858.16 |
| 10 | 8 | 37 | 0.6 | 0.8 | Origami | 4.26E+05 | 232163.926 | 527076 |
| 14 | 24 | 23 | 0.6 | 0.4 | Origami | 3.28E+06 | 4.11E+06 | 3578907.24 |
| 22 | 24 | 37 | 0.6 | 0.4 | Origami | 1.73E+06 | 1.72E+06 | 2234965.2 |
| 25 | 8 | 23 | 0.6 | 0.4 | Origami | 1.04E+06 | 1.21E+06 | 1214800.752 |
| 28 | 24 | 23 | 0.6 | 0.4 | Origami | 4.13E+06 | 4.11E+06 | 3578907.24 |
| 34 | 16 | 30 | 0.7 | 0.6 | Origami | 1.02E+06 | 1.40E+06 | 1407734.79 |
| 38 | 24 | 37 | 0.6 | 0.8 | Origami | 6.13E+05 | 616377.075 | 992832.8881 |
| 40 | 16 | 44 | 0.7 | 0.6 | Origami | 7.44E+05 | 793483.748 | 635448.1225 |
| 41 | 16 | 30 | 0.7 | 0.6 | Origami | 1.60E+06 | 1.40E+06 | 1407734.79 |
| 45 | 24 | 23 | 0.8 | 0.8 | Origami | 1.25E+06 | 1.18E+06 | 1303912.772 |
| 47 | 8 | 37 | 0.8 | 0.8 | Origami | 9.19E+05 | 839182.576 | 954841.6656 |
| 53 | 16 | 30 | 0.5 | 0.6 | Origami | 3.15E+06 | 1.84E+06 | 2469329.388 |
| 54 | 24 | 37 | 0.8 | 0.8 | Origami | 6.85E+05 | 881097.707 | 809154.2209 |
| 72 | 24 | 37 | 0.8 | 0.4 | Origami | 1.10E+06 | 1.11E+06 | 1433623.076 |
| 74 | 16 | 30 | 0.9 | 0.6 | Origami | 1.97E+06 | 1.75E+06 | 1615669.788 |
| 75 | 8 | 37 | 0.6 | 0.8 | Origami | 5.70E+05 | 232163.926 | 527076 |
| 76 | 16 | 16 | 0.7 | 0.6 | Origami | 1.24E+06 | 1.25E+06 | 1533089.712 |
| 77 | 8 | 23 | 0.8 | 0.8 | Origami | 576138 | 610736.156 | 652185.4564 |
| 89 | 24 | 37 | 0.6 | 0.8 | Origami | 9.97E+05 | 616377.075 | 992832.8881 |
| 95 | 8 | 23 | 0.6 | 0.8 | Origami | 1.08E+06 | 1.15E+06 | 1048678.403 |
| 98 | 24 | 37 | 0.6 | 0.4 | Origami | 1.65E+06 | 1.72E+06 | 1641447.816 |
| 103 | 8 | 23 | 0.8 | 0.8 | Origami | 635033 | 610736.156 | 652185.4564 |
| 105 | 16 | 30 | 0.7 | 1 | Origami | 1.28E+06 | 1.28E+06 | 1144985.602 |
| 107 | 16 | 30 | 0.7 | 0.6 | Origami | 1.11E+06 | 1.40E+06 | 1407734.79 |
| 108 | 16 | 30 | 0.7 | 0.6 | Origami | 2.58E+06 | 1.40E+06 | 1407734.79 |
| 110 | 8 | 37 | 0.8 | 0.4 | Origami | 1.19E+06 | 1.41E+06 | 1363546.644 |
| 113 | 32 | 30 | 0.7 | 0.6 | Origami | 1.57E+06 | 1.49E+06 | 1379544.212 |
| 121 | 16 | 30 | 0.7 | 0.6 | Origami | 1.49E+06 | 1.40E+06 | 1407734.79 |
| 128 | 16 | 30 | 0.7 | 0.6 | Origami | 1.25E+06 | 1.40E+06 | 1407734.79 |
| 131 | 24 | 23 | 0.8 | 0.4 | Origami | 1.53E+06 | 1.54E+06 | 1796457.702 |
| 133 | 16 | 16 | 0.7 | 0.6 | Origami | 1.59E+06 | 1.25E+06 | 1533089.712 |
| 134 | 24 | 37 | 0.8 | 0.4 | Origami | 1.38E+06 | 1.11E+06 | 1433623.076 |
| 138 | 16 | 30 | 0.7 | 0.6 | Origami | 1.77E+06 | 1.40E+06 | 1407734.79 |
| 144 | 8 | 37 | 0.6 | 0.4 | Origami | 8.41E+05 | 853550.91 | 816330.3201 |
| 147 | 8 | 37 | 0.8 | 0.8 | Origami | 7.58E+05 | 839182.576 | 954841.6656 |
| 152 | 8 | 23 | 0.6 | 0.4 | Origami | 1.19E+06 | 1.21E+06 | 1214800.752 |
| 154 | 8 | 23 | 0.8 | 0.4 | Origami | 814865 | 937954.965 | 807733.5876 |
| 158 | 16 | 30 | 0.7 | 0.6 | Origami | 6.80E+05 | 1400464.47 | 1407734.79 |
| 160 | 16 | 30 | 0.7 | 0.6 | Origami | 1.02E+06 | 1.40E+06 | 1407734.79 |
| 161 | 32 | 30 | 0.7 | 0.6 | Origami | 1.40E+06 | 1.49E+06 | 1379544.212 |
| 167 | 8 | 23 | 0.8 | 0.4 | Origami | 1.08E+06 | 9.38E+05 | 807733.5876 |
| 168 | 16 | 44 | 0.7 | 0.6 | Origami | 8.48E+05 | 793483.748 | 635448.1225 |
| 170 | 16 | 30 | 0.7 | 0.6 | Origami | 1.73E+06 | 1.49E+06 | 1407734.79 |
| 178 | 8 | 37 | 0.6 | 0.4 | Origami | 8.73E+05 | 853550.91 | 816330.3201 |
| 181 | 24 | 23 | 0.6 | 0.8 | Origami | 2.74E+06 | 2.99E+06 | 2911835.088 |
| 197 | 24 | 23 | 0.8 | 0.4 | Origami | 2.15E+06 | 1.54E+06 | 1796457.702 |
| 202 | 16 | 30 | 0.5 | 0.6 | Origami | 1.83E+06 | 1.84E+06 | 2469329.388 |
| 203 | 24 | 23 | 0.8 | 0.8 | Origami | 1.11E+06 | 1.18E+06 | 1303912.772 |
| 204 | 24 | 23 | 0.6 | 0.8 | Origami | 3.24E+06 | 2.99E+06 | 2911835.088 |
| 207 | 16 | 30 | 0.7 | 0.2 | Origami | 2.08E+06 | 2.04E+06 | 2090858.16 |
| 212 | 16 | 30 | 0.7 | 0.6 | Origami | 1.52E+06 | 1.49E+06 | 1407734.79 |
| 219 | 16 | 30 | 0.9 | 0.6 | Origami | 1.54E+06 | 1.75E+06 | 1615669.788 |
| 220 | 8 | 23 | 0.6 | 0.8 | Origami | 1.24E+06 | 1.15E+06 | 1048678.403 |
| 227 | 8 | 37 | 0.8 | 0.4 | Origami | 1.40E+06 | 1.41E+06 | 1363546.644 |
| 230 | 16 | 30 | 0.7 | 0.6 | Origami | 1.57E+06 | 1.49E+06 | 1407734.79 |
| 6 | 24 | 37 | 0.8 | 0.4 | SHuffle | 1.69E+06 | 1.71E+06 | 1590247.103 |
| 7 | 16 | 30 | 0.7 | 0.6 | SHuffle | 1.60E+06 | 1.71E+06 | 1627767.706 |
| 9 | 24 | 23 | 0.8 | 0.4 | SHuffle | 1.88E+06 | 1.88E+06 | 1961904.462 |
| 19 | 16 | 44 | 0.7 | 0.6 | SHuffle | 1.73E+06 | 1.82E+06 | 2236879.184 |
| 20 | 8 | 37 | 0.6 | 0.8 | SHuffle | 2.45E+06 | 2.60E+06 | 2512985.858 |
| 24 | 24 | 23 | 0.6 | 0.4 | SHuffle | 1.34E+06 | 1.34E+06 | 1405196.868 |
| 27 | 8 | 37 | 0.8 | 0.8 | SHuffle | 9.43E+05 | 1110183.01 | 1352452.703 |
| 31 | 16 | 30 | 0.7 | 0.6 | SHuffle | 1.08E+06 | 1.71E+06 | 1627767.706 |
| 36 | 8 | 37 | 0.8 | 0.4 | SHuffle | 2.61E+06 | 2.10E+06 | 2057675.492 |
| 42 | 16 | 30 | 0.7 | 0.2 | SHuffle | 2.94E+06 | 3.28E+06 | 3363005.823 |
| 43 | 32 | 30 | 0.7 | 0.6 | SHuffle | 3.24E+05 | 288501.494 | 255924.6921 |
| 49 | 8 | 23 | 0.8 | 0.4 | SHuffle | 1.61E+06 | 1.49E+06 | 1552939.669 |
| 59 | 16 | 30 | 0.7 | 0.6 | SHuffle | 1.95E+06 | 1.71E+06 | 1627767.706 |
| 60 | 24 | 23 | 0.8 | 0.8 | SHuffle | 5.18E+05 | 524140.857 | 556859.2129 |
| 70 | 16 | 30 | 0.5 | 0.6 | SHuffle | 1.41E+06 | 1.40E+06 | 1392990.063 |
| 80 | 16 | 30 | 0.7 | 1 | SHuffle | 1.95E+06 | 1.90E+06 | 1676429.353 |
| 88 | 24 | 37 | 0.6 | 0.8 | SHuffle | 1.10E+06 | 1.38E+06 | 1208438.504 |
| 93 | 24 | 37 | 0.6 | 0.4 | SHuffle | 1.66E+06 | 1.67E+**06** | 1983083.568 |
| 96 | 32 | 30 | 0.7 | 0.6 | SHuffle | 2.27E+05 | 288501.494 | 255924.6921 |
| 99 | 16 | 30 | 0.7 | 0.6 | SHuffle | 1.76E+06 | 1.71E+06 | 1627767.706 |
| 104 | 24 | 23 | 0.6 | 0.8 | SHuffle | 6.28E+05 | 681084.402 | 842448.6225 |
| 109 | 16 | 30 | 0.9 | 0.6 | SHuffle | 9.06E+05 | 1038276.33 | 947157.1684 |
| 112 | 24 | 23 | 0.6 | 0.8 | SHuffle | 7.24E+05 | 681084.402 | 842448.6225 |
| 116 | 8 | 37 | 0.6 | 0.4 | SHuffle | 2.15E+06 | 2.33E+06 | 2160488.42 |
| 119 | 16 | 16 | 0.7 | 0.6 | SHuffle | 1.36E+06 | 9.78E+05 | 1267628.292 |
| 123 | 24 | 23 | 0.8 | 0.4 | SHuffle | 1.87E+06 | 1.88E+06 | 1961904.462 |
| 129 | 8 | 23 | 0.8 | 0.8 | SHuffle | 1.21E+06 | 1.02E+06 | 1032296.64 |
| 136 | 16 | 30 | 0.7 | 0.6 | SHuffle | 1.22E+06 | 1.71E+06 | 1627767.706 |
| 137 | 16 | 16 | 0.7 | 0.6 | SHuffle | 1.53E+06 | 9.78E+05 | 1961904.462 |
| 139 | 16 | 30 | 0.7 | 0.6 | SHuffle | 1.19E+06 | 1.71E+06 | 1032296.64 |
| 140 | 16 | 30 | 0.7 | 1 | SHuffle | 1.86E+06 | 1.90E+06 | 1627767.706 |
| 149 | 16 | 44 | 0.7 | 0.6 | SHuffle | 2.43E+06 | 1.82E+06 | 2236879.184 |
| 150 | 24 | 23 | 0.6 | 0.4 | SHuffle | 2.01E+06 | 1.34E+06 | 1405196.868 |
| 156 | 8 | 23 | 0.6 | 0.8 | SHuffle | 1.13E+06 | 1.04E+06 | 1157517.774 |
| 163 | 16 | 30 | 0.7 | 0.6 | SHuffle | 1.66E+06 | 1.71E+06 | 1627767.706 |
| 165 | 8 | 37 | 0.6 | 0.4 | SHuffle | 2.50E+06 | 2.33E+06 | 2160488.42 |
| 166 | 24 | 37 | 0.8 | 0.4 | SHuffle | 1.60E+06 | 1.65E+06 | 1590247.103 |
| 169 | 24 | 37 | 0.8 | 0.8 | SHuffle | 4.57E+05 | 1185986.76 | 319484.9529 |
| 171 | 16 | 30 | 0.7 | 0.6 | SHuffle | 2.40E+06 | 1710164.78 | 1627767.706 |
| 172 | 16 | 30 | 0.7 | 0.6 | SHuffle | 1.62E+06 | 1710164.78 | 1627767.706 |
| 180 | 16 | 30 | 0.9 | 0.6 | SHuffle | 1.05E+06 | 1038276.33 | 947157.1684 |
| 182 | 8 | 37 | 0.8 | 0.8 | SHuffle | 1.26E+06 | 1110183.01 | 1352452.703 |
| 186 | 8 | 23 | 0.6 | 0.4 | SHuffle | 796183 | 760747.538 | 844799.9569 |
| 187 | 16 | 30 | 0.7 | 0.6 | SHuffle | 1.07E+06 | 1710164.78 | 1627767.706 |
| 188 | 8 | 23 | 0.8 | 0.8 | SHuffle | 1.02E+06 | 1016186.71 | 1032296.64 |
| 190 | 8 | 37 | 0.6 | 0.8 | SHuffle | 2.76E+06 | 2602049.62 | 2512985.858 |
| 200 | 16 | 30 | 0.7 | 0.6 | SHuffle | 2.31E+06 | 1710164.78 | 1627767.706 |
| 201 | 24 | 37 | 0.8 | 0.8 | SHuffle | 299200 | 1185986.76 | 319484.9529 |
| 208 | 8 | 23 | 0.8 | 0.4 | SHuffle | 1.36E+06 | 1487933.79 | 1552939.669 |
| 210 | 24 | 37 | 0.6 | 0.8 | SHuffle | 1.39E+06 | 1383871.83 | 1208438.504 |
| 213 | 24 | 23 | 0.8 | 0.8 | SHuffle | 406755 | 524140.857 | 556859.2129 |
| 216 | 16 | 30 | 0.7 | 0.6 | SHuffle | 2.03E+06 | 1710164.78 | 1627767.706 |
| 218 | 24 | 37 | 0.6 | 0.4 | SHuffle | 2.09E+06 | 1669955.74 | 1983083.568 |
| 221 | 8 | 23 | 0.6 | 0.4 | SHuffle | 716805 | 760747.538 | 844799.9569 |
| 222 | 8 | 23 | 0.6 | 0.8 | SHuffle | 1.04E+06 | 1036728.78 | 1157517.774 |
| 228 | 16 | 30 | 0.5 | 0.6 | SHuffle | 1.39E+06 | 1400528.32 | 1392990.063 |
| 231 | 16 | 30 | 0.7 | 0.2 | SHuffle | 3.30E+06 | 3277014.17 | 3363005.823 |
| 232 | 8 | 37 | 0.8 | 0.4 | SHuffle | 2.07E+06 | 2103593.21 | 2057675.492 |
| 3 | 16 | 30 | 0.7 | 0.6 | BL21 | 994240 | 952480.559 | 938961 |
| 5 | 24 | 23 | 0.8 | 0.4 | BL21 | 1.47E+06 | 1280893.3 | 1470835.328 |
| 15 | 16 | 16 | 0.7 | 0.6 | BL21 | 614329 | 637203.546 | 487622.89 |
| 18 | 16 | 30 | 0.7 | 1 | BL21 | 1.36E+06 | 1192875.48 | 1147576.563 |
| 23 | 8 | 23 | 0.8 | 0.4 | BL21 | 1.92E+06 | 1626400.74 | 2234965.2 |
| 35 | 8 | 23 | 0.6 | 0.8 | BL21 | 842346 | 778853.121 | 925905.8176 |
| 37 | 16 | 44 | 0.7 | 0.6 | BL21 | 298877 | 315742.341 | 315271.0201 |
| 39 | 24 | 23 | 0.6 | 0.4 | BL21 | 1.47E+06 | 1739100.54 | 1688024.578 |
| 48 | 16 | 30 | 0.7 | 0.6 | BL21 | 566092 | 952480.559 | 938961 |
| 50 | 24 | 23 | 0.8 | 0.8 | BL21 | 601692 | 629764.558 | 726705.1009 |
| 52 | 24 | 23 | 0.6 | 0.8 | BL21 | 1.71E+06 | 1581936.76 | 1820475.563 |
| 62 | 32 | 30 | 0.7 | 0.6 | BL21 | 2.63E+06 | 2802156.41 | 2601736.74 |
| 64 | 8 | 37 | 0.8 | 0.8 | BL21 | 742854 | 746402.34 | 1008658.662 |
| 65 | 16 | 30 | 0.7 | 0.2 | BL21 | 1.16E+06 | 1446888.96 | 1299577.2 |
| 66 | 16 | 30 | 0.5 | 0.6 | BL21 | 1.70E+06 | 1691600.23 | 1731198.063 |
| 69 | 16 | 30 | 0.9 | 0.6 | BL21 | 1.97E+06 | 1170337.22 | 1666706.82 |
| 73 | 16 | 30 | 0.7 | 0.6 | BL21 | 574711 | 952480.559 | 938961 |
| 81 | 24 | 37 | 0.6 | 0.4 | BL21 | 981855 | 1079543.48 | 1111590.662 |
| 82 | 8 | 37 | 0.6 | 0.4 | BL21 | 358379 | 409748.857 | 423827.0404 |
| 84 | 24 | 37 | 0.8 | 0.4 | BL21 | 1.05E+06 | 1204322.75 | 1200361.272 |
| 92 | 24 | 37 | 0.6 | 0.4 | BL21 | 1.16E+06 | 1079543.48 | 1111590.662 |
| 97 | 24 | 37 | 0.8 | 0.4 | BL21 | 1.36E+06 | 1204322.75 | 1200361.272 |
| 100 | 24 | 37 | 0.6 | 0.8 | BL21 | 3.03E+06 | 2617490.19 | 2510164.923 |
| 102 | 16 | 16 | 0.7 | 0.6 | BL21 | 669722 | 637203.546 | 487622.89 |
| 114 | 24 | 37 | 0.8 | 0.8 | BL21 | 1.18E+06 | 1185986.76 | 1477002.702 |
| 115 | 8 | 37 | 0.6 | 0.8 | BL21 | 838687 | 842196.26 | 888494.76 |
| 118 | 8 | 23 | 0.8 | 0.4 | BL21 | 1.63E+06 | 1626400.74 | 2234965.2 |
| 122 | 8 | 23 | 0.6 | 0.4 | BL21 | 1.55E+06 | 1594878.22 | 1324064.462 |
| 125 | 24 | 37 | 0.6 | 0.8 | BL21 | 2.63E+06 | 2617490.19 | 2510164.923 |
| 126 | 16 | 30 | 0.7 | 0.6 | BL21 | 700796 | 952480.559 | 938961 |
| 127 | 8 | 23 | 0.6 | 0.8 | BL21 | 762372 | 778853.121 | 925905.8176 |
| 132 | 8 | 23 | 0.8 | 0.8 | BL21 | 903343 | 800849.341 | 803192.3641 |
| 141 | 16 | 30 | 0.7 | 0.6 | BL21 | 1.36E+06 | 952480.559 | 1676429.353 |
| 143 | 8 | 37 | 0.8 | 0.4 | BL21 | 1.58E+06 | 1271593.18 | 1261286.225 |
| 146 | 32 | 30 | 0.7 | 0.6 | BL21 | 2.98E+06 | 2802156.41 | 2601736.74 |
| 148 | 16 | 30 | 0.7 | 0.6 | BL21 | 747343 | 952480.559 | 938961 |
| 155 | 24 | 23 | 0.6 | 0.4 | BL21 | 1.92E+06 | 1739100.54 | 1688024.578 |
| 159 | 16 | 30 | 0.7 | 0.6 | BL21 | 1.23E+06 | 952480.559 | 938961 |
| 162 | 24 | 37 | 0.8 | 0.8 | BL21 | 1.33E+06 | 1185986.76 | 1477002.702 |
| 177 | 8 | 37 | 0.6 | 0.8 | BL21 | 1.13E+06 | 1185986.76 | 888494.76 |
| 184 | 8 | 37 | 0.8 | 0.4 | BL21 | 1.27E+06 | 1271593.18 | 1261286.225 |
| 185 | 16 | 30 | 0.7 | 0.6 | BL21 | 516989 | 952480.559 | 938961 |
| 189 | 24 | 23 | 0.8 | 0.4 | BL21 | 1.28E+06 | 1280893.3 | 1470835.328 |
| 191 | 16 | 30 | 0.9 | 0.6 | BL21 | 2.10E+06 | 1170337.22 | 1666706.82 |
| 192 | 8 | 37 | 0.8 | 0.8 | BL21 | 1.03E+06 | 746402.34 | 1008658.662 |
| 193 | 8 | 23 | 0.6 | 0.4 | BL21 | 1.59E+06 | 1594878.22 | 1324064.462 |
| 194 | 16 | 30 | 0.7 | 0.2 | BL21 | 1.45E+06 | 1446888.96 | 1299577.2 |
| 195 | 16 | 30 | 0.7 | 0.6 | BL21 | 1.29E+06 | 952480.559 | 938961 |
| 196 | 16 | 44 | 0.7 | 0.6 | BL21 | 242797 | 315742.341 | 315271.0201 |
| 198 | 16 | 30 | 0.7 | 0.6 | BL21 | 1.52E+06 | 952480.559 | 938961 |
| 199 | 16 | 30 | 0.5 | 0.6 | BL21 | 1.39E+06 | 1691600.23 | 1731198.063 |
| 205 | 8 | 37 | 0.6 | 0.4 | BL21 | 474723 | 409748.857 | 423827.0404 |
| 206 | 24 | 23 | 0.8 | 0.8 | BL21 | 655697 | 629764.558 | 726705.1009 |
| 209 | 8 | 23 | 0.8 | 0.8 | BL21 | 800278 | 800849.341 | 803192.3641 |
| 214 | 16 | 30 | 0.7 | 0.6 | BL21 | 650964 | 952480.559 | 938961 |
| 215 | 24 | 23 | 0.6 | 0.8 | BL21 | 1.60E+06 | 1581936.76 | 1820475.563 |
| 225 | 16 | 30 | 0.7 | 0.6 | BL21 | 1.56E+06 | 952480.559 | 938961 |
| 226 | 16 | 30 | 0.7 | 1 | BL21 | 1.20E+06 | 1192875.48 | 1147576.563 |
